# Prevalence and factors associated with regular fast-food consumption among adults in the UAE: a cross-sectional study

**DOI:** 10.1017/S1368980025101560

**Published:** 2025-12-16

**Authors:** Ala Al Rajabi, Rafiq Hijazi, Lynne Alexandra Kennedy

**Affiliations:** 1 Department of Nutrition Sciences, College of Health Science, QU Health, Qatar University, Doha, Qatar; 2 Department of Mathematics and Statistics, College of Natural and Health Sciences, Zayed University, Abu Dhabi, UAE; 3 Department of Health Sciences, College of Natural and Health Sciences, Zayed University, Abu Dhabi, UAE

**Keywords:** Fast food, Regular fast-food consumption, UAE, Obesity, Public health, Dietary habits, Non-communicable diseases

## Abstract

**Objectives:**

To assess the prevalence and identify sociodemographic predictors of regular fast-food consumption (R-FFC) among United Arab Emirates (UAE) adults to inform public health nutrition responses to growing diet-related ill health in the region.

**Design:**

This is a descriptive cross-sectional study using purposive, convenience sampling. Data were collected using an online survey adapted from two validated surveys and distributed via social media platforms. R-FFC was defined as visiting a fast-food restaurant to eat ≥ 2 times/week. Pearson’s *χ* ^2^ tests and multiple binary logistic regression models were used to investigate prevalence and predictors of R-FFC. All statistical significance was considered at *P*-value < 0·05.

**Setting:**

Community, adults living in the UAE.

**Participants:**

UAE residents, ≥ 18 years, who consumed fast food ≥ once during the previous month.

**Results:**

Totally, 320 respondents met the inclusion criteria (age = mean 23·7 (sd 7·7) years). The prevalence of R-FFC was 46·6 %. Based on results from the regression model, predictors of R-FFC were being female (AOR 2·47; 95 % CI 1·06, 5·79), married (AOR 3·11; 95 % CI 1·25, 7·77), BMI ≥ 25·0 (AOR 2·09; 95 % CI 1·10, 4·00) and residing outside Abu Dhabi (AOR 32·79; 95 % CI 12·06, 89·16). None of the remaining variables reached statistical significance. Taste was the most common reason for FFC (56·9 %), followed by convenience (21·6 %). Regular fast-food consumers were more likely to ‘super-size’ meals (*P* = 0·011), eat alone (*P* = 0·009) and not have regular meal patterns (*P* = 0·004).

**Conclusions:**

The study revealed a high prevalence of R-FFC among UAE adults, and novel cultural predictors and characteristics of FFC in this context, highlighting the importance of socially and culturally informed research and public health strategies in this region.

The United Arab Emirates (UAE) has a population of approximately 10 million and is among the wealthiest countries in the Gulf Cooperation Council (GCC) and the world. Since the regional oil boom of the 1970s, both country and region have experienced considerable social and economic development accompanied by rapid urbanisation, resulting in major environmental and lifestyle changes over a relatively short time. Western-style food retailing, hospitality and fast-food restaurants have become widespread in the past two decades. Fast-food restaurants are outlets where customers typically order and pay before eating. They serve fast food that is convenient, inexpensive and often high in calories, saturated fat, trans fat, sugar, refined carbohydrates and Na^(1)^. In parallel, traditional foods and dietary patterns have been displaced by more socially desirable westernised diets, high in energy, saturated fats, salt, sugars, refined, processed, pre-prepared and fast foods^(2,3)^. In 2018, the first UAE national nutrition and health survey confirmed this shift^(4)^, a finding supported by global reports of continued unhealthy dietary patterns and increasing diet-related risks such as obesity^(5)^. Regular fast-food consumption (R-FFC) is especially problematic since fast food is high in calories, saturated and trans fats, sugars and salt, negatively impacting population health. Fast food (e.g. pizzas, burgers, fried chicken and fries) has become pervasive in the region, replacing more traditional diets. Despite this rapid nutrition transition, few dietary intake studies have been conducted in the UAE. Consequently, nutrition policies and practices are typically informed by the West, and trends are frequently extrapolated from across the GCC region or the broader Middle East.

In response to the rapidly changing food environment and national diet, the prevalence of obesity and interrelated co-morbidities in the UAE population has dramatically risen, in excess of global trends^(6)^. The UAE and its neighbours – Saudi Arabia, Qatar, Bahrain and Kuwait – report among the highest prevalence of obesity globally. The current age-standardised prevalence of obesity among UAE adults is 32·1 % compared with 18·9 % in 1990^(6)^, approximately a 1·7-fold increase over three decades. Strong correlations exist between weight gain, higher BMI and obesity with higher overall food and energy intake, particularly energy-dense foods, including sweetened soft beverages (SSB), and processed, fried and fast foods^(1,7–9)^. Moreover, R-FFC has been directly linked to increased calorie intake, BMI, weight gain and risk of obesity among adults and children^(9–11)^.

The rapid pace of nutrition transition in the UAE is not only linked with increasing obesity but also to a high prevalence of diet-related non-communicable diseases (NCD), including CVD, type 2 diabetes mellitus (T2D) and certain cancers. Western dietary patterns have been linked to increased cardiometabolic risk^(12)^; however, risk varies according to the amount and type of dietary fat^(13)^. There might be a correlation between high trans fatty acid intake and inflammation, oxidative stress and lipo-peroxidation^(14)^, and lowering trans fatty acids may be involved in reducing CVD^(15)^. Furthermore, the literature points to a strong link between fast-food consumption (FFC) and common functional gastrointestinal disorders (FGID)^(16)^.

The aetiology of diet-related obesity and NCD is complex and multifactorial; while genetic factors and energy balance are especially important, structural and societal factors, particularly obesogenic environments, also play an important role^(17–19)^. During the past decade, the fast-food market in the UAE has exploded, posing serious public health concerns. Mixed reports of any association between exposure to fast-food outlets and obesity exist^(20,21)^. As Drewnowski *et al.* warned^(22)^, the impact of built and food environments, such as access to supermarkets, restaurants and fast-food outlets, on dietary choices and obesity remains equivocal. Despite this, little is known about the frequency or characteristics of FFC in the UAE. At the time of writing, this was the first such study. Furthermore, all behaviour, particularly food and eating, is socially and culturally situated. Guided by socioecological principles, FFC is influenced by an interplay of personal, intra- and inter-personal, environmental and contextual factors, including food preferences, taste, convenience and tradition^(23–26)^. Hence, a better understanding of FFC among the population is necessary for planning effective public health policy directed at tackling rising obesity, reducing the individual and societal burden of diet-related diseases and promoting health. This study, therefore, aims to address this gap by assessing prevalence, characteristics and factors associated with R-FFC in the UAE.

## Methods

### Study design and population

A descriptive, cross-sectional study was conducted to assess the prevalence and factors associated with R-FFC among UAE adults. Inclusion criteria were: (1) adults aged ≥ 18 years, (2) current UAE resident, (3) having consumed food from a fast-food restaurant at least once during the past month, (4) holding an active Google account and (5) having provided informed consent to participate before answering the survey. Respondents who did not meet any of the inclusion criteria were excluded from the study. The minimum required sample size was calculated based on a 95 % CI, a 5 % margin of error and an estimated prevalence of R-FFC of about 70 %^(27,28)^. Using a standard sample size formula for proportions^(29)^, the required sample was estimated at 323 participants.

### Data collection instrument

Data collection took place during March 2023, utilising Google Forms online survey. An anonymous survey link was distributed via social media platforms (Twitter, WhatsApp and Snapchat). Purposive, convenient snowball sampling was employed to recruit participants. A single response per participant was ensured via Google Forms settings (limit to a single response). The necessary informed consent, including a brief study description, was obtained from all participants before access to the questionnaire. Participants were informed they could exit the study at any time, without consequence. At the end of the study period, the survey was disabled. The online self-reported survey included a total of thirty-four questions in three sections: sociodemographic characteristics, FFC and the International Physical Activity Questionnaire – Short Form (IPAQ-SF). All survey questions were provided in English only (see online supplementary material, Supplemental Table S1). This is consistent with similar studies of FFC reported in the literature^(10,11,27)^.

Questions that assessed the frequency and specifications of FFC were based on two validated surveys used previously outside the UAE. A culturally modified version of the FFC module from the 2005 Michigan Behavioral Risk Factor Survey (MiBRFS)^(11)^ and the Nelson questionnaire^(30)^ was created for the current study. Face validity of the questionnaire was tested on six bilingual students, and the wording of questions was adjusted for cultural specificity (see online supplementary material, Supplemental Table S1). Based on responses to the survey question *‘In the past month, on average, how many times did you go to a fast-food restaurant to eat?’*, participants were classified as regular fast-food consumers if they went to a fast-food restaurant to eat, on average, two or more times/week during the past month, based on validated instrument^(11)^. The wording implies in-person visits only and does not explicitly include food obtained via delivery apps or take-out.

Participants also self-reported their typical daily intake of fruits and vegetables. Reported servings were categorised as < 2 *v*. ≥ 2 servings/d. This threshold was chosen to indicate low intake, as the majority of participants consumed less than the recommended ≥ 5 servings/d. The fruit and vegetable intake question was similar to those used in international dietary surveys and served as a basic indicator of healthy eating habits. This cut-off has been adopted in previous epidemiological studies to indicate low intake^(31)^. In addition, participants self-reported their current height and weight. BMI was calculated as weight in kilograms divided by height in metres squared (kg/m^2^), and participants were categorised according to WHO criteria.

Self-reported physical activity (PA) during the last 7 day was assessed using the validated IPAQ-SF^(32)^. Data were collected on the weekly frequency and duration of time spent engaging in moderate and vigorous PA, and a total PA estimate was then calculated, expressed as Metabolic Equivalent of Task (MET) minutes of PA a week (MET-min/week)^(32)^. PA was categorised as low (< 600 MET-min/week), moderate (600 ≥ to < 1200 MET-min/week) and high (≥ 1200 MET-min/week)^(33)^.

### Statistical analysis

The sociodemographic characteristics of participants are presented as count (percentage). Prevalence estimates with 95 % CI of R-FFC were calculated for all participants and by the following sociodemographic and health-related characteristics: sex (men; women), age in years (18–24; 25–56), nationality (Emirati; other nationalities), Emirate of residence (Abu Dhabi; others), BMI (underweight/normal weight; overweight/obese), marital status (married/living with a partner; single), the highest level of education (less than bachelor’s degree; bachelor’s degree or higher), employment status (employed; unemployed), general health (poor/fair; good/very good; excellent), PA level (low; moderate-to-high), smoking status (current smoker; former smoker/never-smoker) and fruit and vegetable consumption (< 2 servings/d; ≥ 2 servings/d). Pearson’s *χ* ^2^ test was used to test the association between R-FFC and different study variables. In addition, to investigate further the associations between R-FFC and potential predictors, a multiple binary logistic regression model was performed, adjusted for all socio-economic and health-related variables with FFC (R-FFC *v*. non-regular (NR-FFC)) as the dependent variable. The estimated associations are presented as adjusted OR (AOR) and 95 % CI. Model significance was evaluated using the likelihood ratio test, while model fit was assessed via the Hosmer–Lemeshow test. Predictive performance was measured using receiver operating characteristic (ROC) curve analysis, with the AUC used to quantify accuracy. Multicollinearity was examined through variance inflation factors (VIF), and theoretically relevant interaction terms were tested.

Behavioural characteristics of FFC are presented as prevalence estimates with 95 % CI for all participants and as count (percentage) for regular *v*. non-regular fast-food consumers. Comparisons between regular and non-regular fast-food consumers were conducted using two-proportion *z*-tests. All analyses were performed using SPSS statistical software (SPSS 29.0). All statistical significance was considered at *P*-value < 0·05.

## Results

A total of 343 responses were received. Responders were excluded if they reported not eating from fast-food restaurants during the previous month (*n* 12), resided outside the UAE (*n* 10) or did not provide consent (*n* 1). In total, 320 participants met the inclusion criteria and were included in the final analyses. The target population comprised all adult fast-food consumers in the UAE; however, an online convenience sampling strategy was used, and therefore, the sample may over-represent younger, social-media-savvy individuals. The average age was mean 23·7 (sd 7·7) years. As shown in Table 1, most of the participants were women (80·6 %), Emirati (90·3 %), Middle Eastern (95·0 %), residents of Abu Dhabi (78·8 %), aged 18–24 (79·1 %), single/never married (80·9 %), students (65·0 %), normal or underweight (67·8 %) and never-smokers (87·5 %). Additionally, over half of the participants consumed < 2 servings of fruit and vegetables daily (57·5 %) and reported low PA (57·8 %), although the majority (85·4 %) still rated their general health as ‘good’, ‘very good’ or ‘excellent’.

**Table 1.** Sociodemographic and health-related characteristics of the study population (*n* 320)

	Count	Percentage
Sex		
Women	258	80·6 %
Men	62	19·4 %
Age (years)		
18–24	253	79·1 %
25–34	37	11·6 %
35–44	13	4·1 %
45–56	17	5·3 %
Nationality		
Emirati	289	90·3 %
Other nationalities	31	9·7 %
Ethnicity		
Middle Eastern	304	95·0 %
Far East Asian	2	0·6 %
Southeast Asian	7	2·2 %
African	4	1·2 %
Caucasian/White	3	0·9 %
Emirate of residence		
Abu Dhabi	252	78·8 %
Dubai	27	8·4 %
Sharjah	22	6·9 %
Ajman	10	3·1 %
Fujairah	1	0·3 %
Umm Al-Quwain	1	0·3 %
Ras Al-Khaimah	7	2·2 %
BMI (kg/m^2^)		
Underweight (< 18·5)	48	15·0 %
Normal weight (≥ 18·5 and < 25·0)	169	52·8 %
Overweight (≥ 25·0 and < 30·0)	70	21·9 %
Obese (≥ 30·0)	33	10·3 %
Marital status		
Married/living with a partner	51	15·9 %
Divorced	6	1·9 %
Widowed	1	0·3 %
Separated	3	0·9 %
Single/never married	259	80·9 %
Education		
Less than high school	10	3·1 %
High School	149	46·6 %
Associate degree/diploma	22	6·9 %
Bachelor’s degree	119	37·2 %
Graduate degree (MSc, MBA, PhD and MD)	20	6·3 %
Employment status		
Employed	71	22·2 %
Student	208	65·0 %
Unemployed	39	12·2 %
Retired	2	0·6 %
General health		
Excellent	47	14·7 %
Very good	110	34·4 %
Good	116	36·3 %
Fair	36	11·3 %
Poor	11	3·4 %
Household income (AED/month)		
< 10 000	62	19·4 %
10 000–40 000	71	22·2 %
40 000–80 000	40	12·5 %
> 80 000	24	7·5 %
I prefer not to say	123	38·4 %
Physical activity^*^		
Low	185	57·8 %
Moderate	45	14·1 %
High	90	28·1 %
Smoking status		
Current smoker	21	6·6 %
Former smoker	19	5·9 %
Never-smoker	280	87·5 %
Fruit and vegetable consumption		
< 2 servings/d	184	57·5 %
≥ 2 servings/d	136	42·5 %

* Participants were categorised into low physical activity (< 600 total MET-min/week), moderate physical activity (≥ 600 and < 1200 total MET-min/week) and high physical activity (≥ 1200 total MET-min/week).

Among participants 9·4 % ate at fast-food restaurants once/month, 30·3 % reported eating at fast-food restaurants 2–3 times/month, 13·8 % ate weekly, 20·6 % twice/week and 19·1 % 3–6 times/week, with 6·9 % eating daily. Participants who ate at fast-food restaurants ≥ 2 times/week were categorised as regular fast-food consumers, according to the criteria used by Anderson *et al.* ^(11)^, with 149 of 320 participants (46·6 %) meeting this definition (Table 2). The prevalence of R-FFC and its associations with different study variables is presented in Table 2. The significance of associations was tested using Pearson’s *χ* ^2^ test. The prevalence of regular fast-food consumers was higher among women compared to men (50·4 % *v*. 30·6 %; *P*-value = 0·005) and among non-Abu Dhabi residents compared to those residing in Abu Dhabi (92·6 % *v*. 34·1 %; *P*-value < 0·001). None of the other variables – including age, nationality, BMI, marital status, education, employment status, self-reported general health, PA, smoking status and fruit and vegetable consumption – reached statistical significance (*P*-value ≥ 0·05).

**Table 2.** Unadjusted prevalence and AOR of regular fast-food consumption^‡^ by sociodemographic and health-related characteristics among study participants (*n* 320)

	Unadjusted prevalence			Regression model^†^		
	%	95 % CI	*P*-value^§^	AOR^||^	95 % CI	*P*-value^¶^
Overall	46·6	41·2, 52·0	NA	NA		NA
Sex						
Women	50·4	44·3, 56·4	**0·005** ^*^	2·47	1·06, 5·79	**0·037** ^*^
Men	30·6	20·2, 42·8		1 (reference)		
Age (years)						
18–24	47·0	40·9, 53·2	0·742	1·26	0·49, 3·25	0·627
25–56	44·8	33·3, 56·7		1 (reference)		
Nationality						
Emirati	47·1	41·4, 52·8	0·587	1·58	0·60, 4·19	0·358
Other nationalities	41·9	26·4, 59·3		1 (reference)		
Emirate of residence						
Abu Dhabi	34·1	28·5, 40·1	**< 0·001** ^*^	1 (reference)		
Others^#^	92·6	84·6, 97·1		32·79	12·06, 89·16	**< 0·001** ^*^
BMI (kg/m^2^)						
Underweight/normal weight (< 25·0)	44·7	38·2, 51·3	0·332	1 (reference)		
Overweight/obese (≥ 25·0)	50·5	40·9, 60·0		2·09	1·10, 4·00	**0·025** ^*^
Marital status						
Married/living with a partner	51·0	37·5, 64·3	0·490	3·11	1·25, 7·77	**0·015** ^*^
Single^††^	45·7	39·8, 51·7		1 (reference)		
Education						
Less than Bachelor’s degree^‡‡^	48·6	41·4, 55·9	0·400	1·21	0·68, 2·15	0·516
Bachelor’s degree or higher^§§^	43·9	35·8, 52·2		1 (reference)		
Employment status						
Employed	43·7	32·6, 55·3	0·579	1 (reference)		
Unemployed^||||^	47·4	41·2, 53·6		1·25	0·52, 3·01	0·613
General health						0·089^##^
Excellent	31·9	20·0, 46·0	0·057	1 (reference)		
Very good/good	50·4	44·0, 56·9		2·28	0·97, 5·38	0·059
Fair/poor	42·6	29·2, 56·8		1·33	0·44, 3·97	0·614
Physical activity^¶¶^						
Low	50·3	43·1, 57·4	0·120	1·43	0·83, 2·48	0·197
Moderate, high	41·5	33·4, 49·9		1 (reference)		
Smoking status						
Current smoker	42·9	23·7, 63·8	0·725	1 (reference)		
Former smoker/never-smoker	46·8	41·2, 52·5		1·13	0·31, 4·13	0·852
Fruit and vegetable consumption						
< 2 servings/d	46·2	39·1, 53·4	0·878	1 (reference)		
≥ 2 servings/d	47·1	38·8, 55·4		1·04	0·60, 1·80	0·890

AOR, adjusted OR.

* Statistically significant.

† The final model was statistically significant (likelihood ratio test: χ² = 108·66, *P* < 0·001) and demonstrated good fit (Hosmer–Lemeshow test: χ² = 7·02, *P* = 0·53). Variance inflation factors (1·06–2·07) indicated no multicollinearity issues. The model showed acceptable discrimination, with an AUC of 0·797 (95 % CI 0·748, 0·846). Theoretically relevant interaction terms (e.g. Gender × Physical Activity, Education × Health) were tested but were not statistically significant (*P* > 0·05); therefore, they were excluded from the final model.

‡ Consumed fast food ≥ 2 times/week based on the question: ‘*In the past month, on average, how many times did you go to a fast-food restaurant to eat?*’.

§ Calculated by *χ* ^2^ test.

|| Adjusted OR from multiple binary logistic regression model with regular fast-food consumption (≥ 2 times/week) as the dependent variable and all demographic, socio-economic and health-related characteristics as the covariates. Non-regular fast-food consumption was assigned as the reference level for the analysis.

¶ Adjusted effect *P*-value.

# Dubai, Sharjah, Ajman, Fujairah, Umm Al-Quwain and Ras Al-Khaimah.

†† Single includes divorced, separated, widowed and single/never married.

‡‡ Associate degree/college diploma, high school diploma or less than a high school diploma.

§§ Bachelor’s degree or graduate degree (MSc, MBA, PhD and MD).

|||| Unemployed includes unemployed, retired and student.

¶¶ Participants were categorised into low physical activity (< 600 total MET-min/week) *v*. moderate-to-high physical activity (≥ 600 total MET-min/week).

## Overall *P*-value.

To further clarify the factors associated with R-FFC, a multiple binary logistic regression model was conducted with the frequency of FFC (regular *v*. non-regular) as the dependent variable. Table 2 provides AOR and the corresponding 95 % CI for the variables included in the model. Results from the regression model showed that – compared to respective reference categories – being female (AOR 2·47; 95 % CI 1·06, 5·79), residing outside Abu Dhabi (AOR 32·79; 95 % CI 12·06, 89·16), BMI ≥ 25·0 (AOR 2·09; 95 % CI 1·10, 4·00) and being married/living with a partner (AOR 3·11; 95 % CI 1·25, 7·77) were all significantly associated with increased odds of R-FFC. None of the remaining model variables achieved statistical significance (Table 2). Additionally, a sub-analysis including only participants who reported household income (*n* 197) found no significant association between income and R-FFC (see online supplementary material, Supplemental Table S2).

The majority of participants (56·9 %) selected the taste of food as the main reason for choosing fast-food restaurants, followed by fast-food restaurants being viewed as quick and convenient (21·6 %), sociability (12·8 %) and its good value in terms of cost (8·8 %) (Table 3). Dinner was the meal most frequently eaten at a fast-food restaurant (selected by 56·3 % of participants), followed by lunch (10·6 %) and then breakfast (4·7 %), while approximately a quarter (26·6 %) of participants reported no specific or usual meal type. The majority of participants usually went with family when visiting fast-food restaurants (52·8 %), and at least half of the time ordered meal packages (74·7 %) and ‘take-out’ (87·2 %) with 64·9 % of ‘take-out’ orders consumed at home. Only 27·8 % of participants (*n* 89) noticed the availability of nutritional information at the restaurants they visited, with the majority (*n* 58; 65·2 %) reporting having read it. Of the fifty-eight respondents who had read nutritional information, the vast majority (*n* 52; 89·7 %) used this information in decisions when ordering, at least half the time. Overall, only 16·3 % (*n* 52/320) of participants used nutritional information when ordering. However, in response to a hypothetical question, 39·1 % of participants said that they would be very or somewhat likely to order ‘healthier’ food items ‘when available’ (Table 3).

**Table 3.** Characteristics of fast-food consumption among study participants (*n* 320)

Characteristic	Fast-food consumption (count (%))						
	Overall		Non-regular^†^ (*n* 171)		Regular^‡^ (*n* 149)		*P*-value^§^
	%	95 % CI	Count	%	Count	%	
Main reason for choosing fast-food restaurants							0·257^††^
Quick, convenient	21·6	17·3, 26·3	30	17·5 %	39	26·2 %	0·061
Taste of the food	56·9	51·4, 62·2	103	60·2 %	79	53·0 %	0·194
Sociability	12·8	9·5, 16·8	24	14·0 %	17	11·4 %	0·483
Value, cost	8·8	6, 12·2	14	8·2 %	14	9·4 %	0·703
Usual meal eaten							**0·035** ^††,^ *
Breakfast/lunch	15·3	11·7, 19·6	28	16·4 % %	21	14·1 %	0·572
Dinner	56·3	50·8, 61·6	106	62·0 %	74	49·7 %	**0·027** ^*^
Snack	1·9	0·8, 3·8	3	1·8 %	3	2·0 %	0·865
All meals, no usual meal	26·6	21·9, 31·6	34	19·9 %	51	34·2 %	**0·004** ^*^
Usual order							0·217^††^
Meal package	56·9	51·4, 62·2	96	56·1 %	86	57·7 %	0·776
Individual items	25·3	20·8, 30·3	49	28·7 %	32	21·5 %	0·141
Each about half the time	17·8	13·9, 22·3	26	15·2 %	31	20·8 %	0·192
‘Super-size’ options are usually ordered							
Yes	21·3	17, 26	27	15·8 %	41	27·5 %	**0·011** ^*^
Eat-in or take-out							0·162^††^
Eat-in	12·8	9·5, 16·8	23	13·5 %	18	12·1 %	0·715
Take-out	57·5	52, 62·8	105	61·4 %	79	53·0 %	0·130
Each about half the time	29·7	24·9, 34·9	43	25·1 %	52	34·9 %	0·057
Where take-out is usually eaten^||^							0·133^††^
In the car/at the office	35·1	29·7, 40·9	46	31·1 %	52	39·7 %	0·133
At home	64·9	59·1, 70·3	102	68·9 %	79	60·3 %	0·133
Usually accompanied by							**0·011** ^††,^ *
Family	52·8	47·3, 58·2	102	59·6 %	67	45·0 %	**0·009** ^*^
Friends/co-workers	28·4	23·7, 33·6	46	26·9 %	45	30·2 %	0·514
By myself	18·8	14·8, 23·3	23	13·5 %	37	24·8 %	**0·009** ^*^
Nutritional information available at restaurant							0·153^††^
Yes	27·8	23·1, 32·9	55	32·2 %	34	22·8 %	0·063
No	21·3	17, 26	36	21·1 %	32	21·5 %	0·926
Never noticed, never looked	50·9	45·5, 56·4	80	46·8 %	83	55·7 %	0·111
Reading nutritional information^¶^							0·148^††^
Yes	65·2	54·9, 74·5	39	70·9 %	19	55·9 %	0·148
No	34·8	25·5, 45·1	16	29·1 %	15	44·1 %	0·148
How often does nutritional information help you decide what to order?^#^							0·735^††^
Always/most of the time	62·1	49·2, 73·7	17	60·7 %	19	63·3 %	0·837
About half the time/sometimes	27·6	17·4, 40·0	9	32·1 %	7	23·3 %	0·453
Never	10·3	4·4, 20·1	2	7·1 %	4	13·3 %	0·439
Ordering healthier food items							0·684^††^
Very likely/somewhat likely	39·1	33·8, 44·5	70	40·9 %	55	36·9 %	0·462
Somewhat unlikely/very unlikely	44·4	39·0, 49·8	75	43·9 %	67	45·0 %	0·842
Neither likely nor unlikely/don’t know	16·6	12·8, 20·9	26	15·2 %	27	18·1 %	0·484

* Statistically significant.

† Consumed fast food < 2 times/week based on the question: ‘*In the past month, on average, how many times did you go to a fast-food restaurant to eat?*’.

‡ Consumed fast food ≥ 2 times/week based on the question: ‘*In the past month, on average, how many times did you go to a fast-food restaurant to eat?*’.

§ Calculated by two-proportion *z*-test comparing regular *v*. non-regular fast-food consumers.

|| Applies to participants whose answer to the previous question was ‘Take-out’ or ‘Each about half the time’ (*n* 279).

¶ Applies to participants who answered yes to the previous question on the availability of nutritional information (*n* 89).

# Applies to participants who answered yes to the previous question on reading nutritional information (*n* 58).

†† Calculated by *χ* ^2^ test of independence for the overall association between characteristic and consumer type.

Higher proportions of regular, compared to non-regular fast-food consumers reported not having a usual meal (34·2 % *v*. 19·9 %; *P*-value = 0·004), usually selecting the ‘super-size’ option (27·5 % *v*. 15·8 %, *P*-value = 0·011) and visiting fast-food restaurants by themselves (24·8 % *v*. 13·5 %, *P*-value = 0·009). Conversely, lower proportions of regular consumers reported dinner as their usual meal (49·7 % *v*. 62·0 %, *P*-value = 0·027) and going to fast-food restaurants with family (45·0 % *v*. 59·6 %, *P*-value = 0·009) (Table 3). Additionally, higher proportions of regular fast-food consumers reported convenience as the main reason for choosing fast-food restaurants (26·2 % *v*. 17·5 %, *P*-value = 0·061), while lower proportions of regular consumers reported the presence of nutritional information at the visited restaurant (22·8 % *v*. 32·2 %; *P*-value = 0·063). However, these trends did not reach statistical significance (Table 3).

Participants reported the type of fast-food restaurants they visited^(30)^ (Table 4). The most frequented were traditional fast-food restaurants (burgers and fries), chosen by 81·9 % of participants. This was followed by fried chicken restaurants (48·1 %), coffee shops (47·2 %), pizza restaurants (41·6 %), sandwich shops (30·3 %), ice cream and burger shops (30·3 %), snack bars (26·3 %) and bakery/donut shops (21·9 %). Surprisingly, only 19·4 % of participants reported visiting traditional Emirati food restaurants (Table 4). When comparing regular fast-food consumers to non-regular consumers, a significantly higher proportion of regular consumers visited fried chicken restaurants (54·4 % *v*. 42·7 %; *P*-value = 0·037). No other comparison between regular and non-regular consumers reached statistical significance.

**Table 4.** Type of fast-food restaurants visited by study participants^^^ (*n* 320)

Characteristic			Fast-food consumption (count (%))				
	Overall		Non-regular^†^ (*n* 171)		Regular^‡^ (*n* 149)		*P*-value^§^
	%	95 % CI	Count	%	Count	%	
Type of fast-food restaurant							
Traditional fast food^||^	81·9	77·4, 85·8	139	81·3 %	123	82·6 %	0·770
Fried chicken^¶^	48·1	42·7, 53·6	73	42·7 %	81	54·4 %	**0·037** *
Mexican fast food^#^	10	7·1, 13·6	12	7·0 %	20	13·4 %	0·057
Sandwich or sub shop^††^	30·3	25·5, 35·5	49	28·7 %	48	32·2 %	0·489
Bakery or donut shop	21·9	17·6, 26·6	33	19·3 %	37	24·8 %	0·232
Ice cream and burger shop	30·3	25·5, 35·5	46	26·9 %	51	34·2 %	0·155
Bagel shop	4·1	2·3, 6·7	4	2·3 %	9	6·0 %	0·094
Coffee shop	47·2	41·8, 52·7	79	46·2 %	72	48·3 %	0·704
Pizza^‡‡^	41·6	36·3, 47	70	40·9 %	63	42·3 %	0·807
Asian	17·5	13·6, 21·9	31	18·1 %	25	16·8 %	0·751
Traditional Emirati food^§§^	19·4	15·3, 24	34	19·9 %	28	18·8 %	0·805
Snack bar^||||^	26·3	21·7, 31·3	44	25·7 %	40	26·8 %	0·821

* Statistically significant.

^ Participants were asked to select all that apply in response to the question: *‘If you went to these types of restaurants in the past month, which restaurants did you go to?’*.

† Consumed fast food < 2 times/week based on the question: ‘*In the past month, on average, how many times did you go to a fast-food restaurant to eat?*’.

‡ Consumed fast food ≥ 2 times/week based on the question: ‘*In the past month, on average, how many times did you go to a fast-food restaurant to eat?*’.

§ Calculated by two-proportion *z*-test comparing regular *v*. non-regular fast-food consumers.

|| Burger-and-fries fast-food restaurants such as McDonalds®, Burger King® and Hardee’s®.

¶ Such as KFC®.

# Such as Taqado® Mexican Chicken.

†† Such as Subway®.

‡‡ Such as Pizza Hut®.

§§ Such as Luqaimat (sweet dumplings).

|||| Such as the ones found inside large hypermarkets, for example, Lulu® and Carrefour®.

## Discussion

This study examined the frequency and characteristics of FFC among UAE adults, and to our knowledge, this is the first study conducted on this population. As illustrated by the findings and using the criterion (≥ twice a week; fast food purchased at restaurants), almost half (46·6 %) of participants were regular fast-food consumers. Of these, 26 % consumed daily or almost daily, while approximately a third (30·3 %) reported more modest occasional FFC. In addition, only 19·4 % of participants visited traditional Emirati food restaurants. These findings highlight a clear shift towards FFC patterns and suggest that fast food and R-FFC, as a non-traditional dietary behaviour, are firmly embedded in Emirati food culture, potentially displacing traditional eating habits.

The UAE food environment has changed substantially over the past two decades; fast-food outlets are now omnipresent, and fast food is both affordable and accessible around the clock. In the current study’s predominantly Emirati sample, income was not significantly associated with R-FFC. Emirati heritage is protected through government financial incentives; therefore, for most Emirati adults, food affordability is unlikely to be a major determinant of food choices or dietary behaviour.

Although inconclusive, research suggests an association between increased accessibility, including the density of fast-food outlets in neighbourhoods, and higher BMI^(34)^. In the UAE and neighbouring GCC countries, rapid urbanisation, vast wealth and high disposable incomes have fuelled an explosion in food imports, retailing and hospitality, including the proliferation of fast-food outlets. The pace of the UAE nutrition transition is relatively unprecedented, reflecting a clear shift from traditional Emirati diets towards Western, and fast, foods^(4)^, supporting anecdotal claims that Emiratis now consume considerably more of these foods. Moreover, market intel research suggests that ‘eating out’ and ‘ordering in’ have become a social pastime in this region^(35)^, a context-specific trend that is worthy of further exploration to understand its contribution to diet-related ill health.

Comparing the prevalence of R-FFC in the UAE with published studies, including those conducted in the GCC and Arab region, proved challenging. The criteria and FFC frequency used to define R-FFC, and thus the range of results varied widely. Furthermore, even within studies using the same criterion as the current study (i.e. ≥ twice/week), prevalence rates for R-FFC varied substantially. For instance, studies reported the prevalence of regular fast-food consumers to be 28 % of adults in Michigan^(11)^, 47·5 % of adults in Saudi Arabia^(27)^, 81·4 % of mostly young (20·99 (sd 3·14) years), female (71 %) in Kuwait^(28)^ and 29·1 % of university students in Syria^(36)^. Studies that used different frequencies to define R-FFC (≥ once/week; ≥ 3 times/week) reported an equally diverse range of regular consumption^(37–40)^. This wide variation might be explained by reporting bias common in self-reported dietary studies^(41)^, where social desirability, and therefore, social acceptability of foods, including fast food, fluctuates over time. The UAE’s rapid nutrition transition has shifted social attitudes towards FFC. Therefore, regardless of the criteria used, the prevalence of R-FFC is both temporal and contextual, supporting calls for socially situated research to inform contextually relevant solutions. Understanding cultural context is crucial for understanding the prevalence and characteristics of lifestyle behaviours like FFC.

### Characteristics of regular fast-food consumers

Regression results revealed that higher BMI, being female, married and residing outside Abu Dhabi were associated with higher odds of R-FFC. Among participants residing outside Abu Dhabi (21 % of the sample, *n* 68), 92·6 % were categorised as regular consumers (63 of 68), compared with 34·1 % among those living in Abu Dhabi. The large AOR for residence (AOR 32·79) is likely inflated by sparse cell counts – only five non-regular consumers outside Abu Dhabi – which can produce unstable estimates in logistic regression. This finding should therefore be interpreted with caution.

Participants with BMI of 25 were twice as likely to regularly consume fast food compared with those with a BMI < 25. These findings align with substantial literature reporting a positive association between body weight and R-FFC, regardless of the criteria used to define R-FFC. A study in Saudi Arabia showed increased odds of R-FFC among overweight and obese participants^(42)^. Furthermore, the Michigan study found that R-FFC was associated with higher BMI, with regular consumers having 60–80 % higher odds of obesity compared to those who ate fast food < once/week^(11)^. Other studies reported that R-FFC was associated with an increased risk of obesity^(10,40,43)^, abdominal obesity^(39)^ and greater weight gain^(13)^. While current study findings are consistent with the existing body of evidence, the relationship between R-FFC and increased BMI remains complex. It is unclear whether this association is primarily driven by FFC itself or by the quantity and quality of the remaining diet^(10,44)^. This could be explained by R-FFC’s link to poor dietary quality and increased energy intake, resulting in weight gain^(10)^. Regular consumers were significantly more likely to order ‘supersize’ items, aligning with Anderson *et al.* ^(11)^. Additionally, studies suggest that regular fast-food consumers may grossly underestimate caloric intake^(45)^, further contributing to the association between R-FFC and increased BMI.

Contrary to most studies, the present study estimated that 50·4 % of women were regular fast-food consumers compared to 30·6 % of men, with women more than twice as likely to be regular consumers after adjustment. This aligns with Barrington *et al.*, who also reported higher FFC among females^(46)^, whereas most studies, conducted on Western and non-Western populations, consistently suggest that men are more frequent consumers^(11,43)^. This anomaly may reflect sociocultural factors in Emirati society: men regularly gather for coffee, while women – constrained by cultural and religious norms – are less likely to visit such venues, and alternatives, including fast-food restaurants, are chosen especially when accompanied by their children or siblings in their care. Furthermore, despite societal developments, the family remains at the centre of Emirati society. Compared with Western trends, Emirati culture still encourages women to marry and have children at a young age. These dynamics may help explain why over half (52 %) of participants consumed fast food with family, compared with only 28·4 % with friends. The sample’s gender imbalance (80·6 % female) may have amplified female-specific patterns; therefore, this association should be interpreted with caution and examined in more balanced samples. Interestingly, unlike studies linking single status to higher FFC^(42,47,48)^, married participants were three times as likely to be regular fast-food consumers. These findings underscore the need to consider gender-specific and cultural factors when analysing FFC patterns in the UAE, while differences in consumption criteria and R-FFC definitions within the literature necessitate careful interpretation.

Taste was the primary motivator for FFC, cited by 56·9 % of study participants, far outweighing convenience (21·6 %), with no significant difference between regular and irregular consumers. Similar findings were reported in an earlier pilot study^(49)^ and in studies conducted across the GCC^(27,28,47)^. However, several other studies identified convenience as the main determinant^(37,50–52)^. For instance, Anderson *et al.* ^(11)^ reported that 63·8 % of adults chose fast food for being ‘quick and convenient’, while only 16·4 % cited taste. Although further research is warranted, this contradiction appears mainly between Arab and Western populations, suggesting sociocultural factors may be involved. In the UAE and across the GCC, housemaids are commonplace and often prepare meals, suggesting convenience may be less influential. Social desirability and palatability of Western and fast foods likely contribute as well. As previously suggested, primordial food preferences (high fat, salt, sugar and energy-dense foods) shape taste preferences and food choices^(8)^, reinforcing the appeal of Western and fast foods over convenience in this context.

Healthy options enable healthier choices. However, only 39·1 % of participants were likely to order healthier food if available, while 44·4 % were unlikely to do so. Providing restaurant nutrition information, particularly calorie labelling schemes, has been shown to encourage healthier food choices^(53)^. Despite this, less than 1 in 5 participants (16·3 %; *n* 52/320) used nutritional information when ordering fast food, consistent with Anderson *et al.* ^(11)^. In 2019, the UAE government launched its nutritional labelling policy, which includes voluntary nutritional and calorie labelling for restaurants with more than twenty outlets^(54)^. Since many fast-food outlets in the UAE are independent or temporary ‘pop-up’ restaurants, we strongly recommend introducing supportive policy measures to mandate calorie labelling for all fast-food outlets. Further research into the characteristics and motivations of FFC in the UAE will help design culturally appropriate interventions and messaging.

### Limitations

The present study is novel and timely as one of the first cross-sectional investigations of R-FFC among UAE adults; however, the relatively small sample size may limit generalisability. The cross-sectional study design precludes causal inference. Furthermore, reliance on self-reported data can potentially introduce recall and social desirability bias, as well as measurement error – for example, self-reported height and weight could affect BMI classification. Additionally, participants were not provided with a clear definition of ‘fast-food restaurants’, which may have led to a misestimation of FFC frequency. Because the R-FFC definition emphasised in-person visits to fast-food restaurants, app-based delivery and take-out orders were not counted. Given high online food delivery use among youth and young adults in the UAE, this likely underestimates the true prevalence of R-FFC,^(55,56)^, which could limit external validity. Despite this, anonymity was ensured to help mitigate biases, and unique sociocultural factors may explain discrepancies with previous research.

Furthermore, conducting an English survey in an Arabic-speaking country could have posed challenges for some participants; however, English is widely spoken, particularly among younger Emiratis, who constituted most of this sample, so this is unlikely to be a major concern. The online survey was distributed via social media channels, and participants self-selected and were recruited through purposive, convenience, and snowball sampling, which is likely to introduce recruitment bias. Notably, most participants were skewed towards young, female and educated adults; therefore, the findings are descriptive of the sample and not generalisable to older adults, less digitally literate individuals, males or the entire UAE adult population.

At a broader systems level, survey research in the UAE – and similar GCC contexts – is shaped by unique social and cultural factors. The absence of a unified postal address system and limited postal services limits systematic postal surveys, leading independent researchers to turn to social media, which, by its nature, fosters convenience sampling. To strengthen causal inference and improve representativeness, future research should adopt longitudinal prospective designs with targeted and randomised or probability recruitment strategies.

While the above limitations may reduce generalisability, in the absence of UAE-specific surveys of FFC, the insights into the estimated frequency and characteristics of UAE R-FFC patterns remain valuable and likely outweigh limitations.

### Implications for policy and practice

In summary, R-FFC is highly prevalent among UAE adults, with distinct sociodemographic and behavioural determinants. These findings reinforce calls for contextualised, culturally informed public health strategies in the UAE and GCC, to reduce fast-food intake, promote healthier diets and help combat obesity and NCD in the region. Future efforts might focus on monitoring NCD trends, identifying behavioural risk factors and informing prevention efforts. Additionally, more funding is needed to support appropriate public health nutrition, nutrition epidemiology and social research that accounts for the unique social and cultural context of this region, including the application of established techniques and the development of innovative methods to support future researchers in designing more reliable and representative online research.

Despite limitations, the results have clear public health implications. The high prevalence of R-FFC suggests the need for multifaceted interventions in the UAE. Policy measures could include mandating clear nutritional labelling in fast-food outlets and restricting portion sizes or ‘super-size’ promotions. Educational campaigns could address the dominance of taste and convenience in food choices by highlighting the benefits and palatability of healthier options. In schools and workplaces, interventions should provide healthy meal options and discourage frequent fast-food visits. Given the importance of social and family influences, community and family-based programmes promoting family mealtimes, and involving parents in nutrition education, might help curb fast-food frequency.

### Conclusion and recommendations

In the UAE and globally, eating at fast-food restaurants has become a popular social activity and lifestyle choice. Research indicates that R-FFC is linked to various health issues and chronic diseases, making it a public health concern. The current study found that nearly half of the participants, especially Emirati women aged 18–24 years, consumed fast-food regularly. Furthermore, regular consumers choose larger meal sizes and eat fast food outside traditional mealtimes, primarily for taste rather than convenience or social reasons. This trend reflects a shift away from traditional food practices, and therefore, policy measures, including regulation of the fast-food environment and mandatory calorie labelling, are needed to mitigate the potential health risks associated with this dietary pattern.

## Supporting information

Al Rajabi et al. supplementary material 1 Al Rajabi et al. supplementary material

Al Rajabi et al. supplementary material 2 Al Rajabi et al. supplementary material

## Supplementary material

For supplementary material accompanying this paper visit https://doi.org/10.1017/S1368980025101560
