# Supplementary material for: Prevalence and factors associated with regular fast-food consumption among adults in the UAE: a cross-sectional study
Source: Public Health Nutr. 2025 Dec 16;29(1):e2. doi: 10.1017/S1368980025101560 (PMC12809606; doi:10.1017/S1368980025101560)
Supplement: Al Rajabi et al. supplementary material 1 — Al Rajabi et al. supplementary material [file S1368980025101560sup001.docx]

**Supplementary Table S1**. Survey questions with their responses.

| **Sections** | **Questions** | **Answer options** |
| --- | --- | --- |
| 1. **Sociodemographic information** | 1. I agree to participate in this study. | - Yes - No ***(Skip to submit)*** |
|  | 1. In the past month, did you eat from any fast-food restaurant? | - Yes - No ***(Skip to submit)*** |
|  | 1. Which Emirate do you live in? | - Abu Dhabi - Dubai - Sharjah - Ajman - Ras Al-Khaimah - Umm Al Quwain - Fujairah - I don’t live In UAE ***(Skip to submit)*** |
|  | 1. What is your age (years)? **(open-ended question)** |  |
|  | 1. What is your weight (kg)? **(open-ended question)** |  |
|  | 1. What is your height (cm)? **(open-ended question)** |  |
|  | 1. Are you an Emirati citizen? | - Yes - No |
|  | 1. What is your gender? | - Female - Male |
|  | 1. What is your ethnicity? | - Middle Eastern (e.g. Arabs, Persians, Turkish, Kurds) - Far East Asian (e.g. Chinese, Pilipino, Japanese, Korean) - Southeast Asian (e.g. Indian, Pakistani, Bengali, Sri Lanka) - African - White/Caucasian |
|  | 1. What is your current marital status? | - Married and/or living with a partner - Divorced - Widowed - Separated - Single, never married |
|  | 1. What is the highest level of education you have completed? | - Less than a high-school diploma - High school diploma - Associate degree/college diploma - Bachelor's degree - Graduate degree (MSc, MBA, PhD, MD) |
|  | 1. What is your current employment status? | - Unemployed - Full-time employee - Part-time employee - Student - Self-employment - Retired |
|  | 1. What is your monthly household income? | - Less than 10,000 AED - 10,000 to 20,000 AED - 20,000 to 40,000 AED - 40,000 to 60,000 AED - 60,000 to 80,000 AED - 80,000 to 100,000 AED - More than 100,000 AED - I prefer not to say |
|  | 1. How would you rate your general health? | - Excellent - Very good - Good - Fair - Poor |
|  | 1. What best describes your current situation regarding cigarette smoking? | - I currently smoke - I used to smoke - I have never smoked |
|  | 1. How often do you consume fruits and vegetables daily? | - 0-1 servings per day - 2-3 servings per day - 4-5 servings per day - More than 5 servings per day |
| 1. **Fast food consumption** | 1. In the past month, on average, how many times did you go to a fast-food restaurant to eat? | - 1 time per month - 2-3 times per month - 1 time per week - 2 times per week - 3-4 times per week - 5-6 times per week - 1 time per day - 2 times per day - 3 times or more per day |
|  | 1. When you go to a fast-food restaurant, what is the main reason you choose this type of restaurant instead of another type? | - Taste of the food, you enjoy going to fast-food restaurants - Value or cost - Convenience, fast service, it’s quick - The person you are with wants to go - Your children like fast-food restaurants - Fast-food restaurants are conveniently located |
|  | 1. When you go to a fast-food restaurant, do you usually eat breakfast, lunch, dinner, or a snack? | - Breakfast - Lunch - Dinner - Snack - All meals, no usual meal |
|  | 1. When you go to a fast-food restaurant, do you usually order a meal-package or individual items? | - Meal Package - Individual items - Each about half the time |
|  | 1. When you go to a fast-food restaurant, do you usually order any of the “super-size” options that are available? | - Yes - No |
|  | 1. When you go to a fast-food restaurant, do you usually eat in the restaurant or take out? | - Eat in ***(Skip to question 24)*** - Take out - Each about half the time |
|  | 1. Where do you usually eat your take-out? | - In the car - At home - At the office |
|  | 1. When you go to a fast-food restaurant, do you usually go with family, friends, co-workers, or by yourself (with whom do you go most often)? | - Family - Friends - Co-workers - By myself |
|  | 1. Sometimes fast-food restaurants have information available about the nutritional contents of their foods. Is this type of nutritional information available at the fast-food restaurants you usually go to? | - Yes - No ***(Skip to question 28)*** - Never noticed, never looked ***(Skip to question 28)*** |
|  | 1. Do you ever read this nutritional information at fast-food restaurants? | - Yes - No ***(Skip to question 28)*** |
|  | 1. How often does this nutritional information help you decide what to order? Would you say . . . | - Always - Most of the time - About half the time - Sometimes - Never |
|  | 1. Some fast-food restaurants are including healthier items on their menu. On a usual basis, how likely are you to order healthier food items? Would you say that you are | - Very likely - Somewhat likely - Somewhat unlikely - Very unlikely - Neither likely nor unlikely - Don't know |
|  | 1. If you went to these types of restaurants in the past month, which restaurants did you go to? **(choose all that apply question)** | - Traditional (burger-and-fries) fast food restaurants, such as McDonalds, Burger King, Hardees - Fried chicken, such as KFC - Mexican fast-food such as Taqado Mexican Chicken - Sandwich or sub shop (like Subway) - Bakery or donut shop - Ice cream and burger shops - Bagel shop - Coffee shop - Pizza restaurants such as Pizza Hut - Asian fast food such as Chinese, Korean, Vietnamese - Traditional Emirati food such as Luqaimat - Snack bars in stores like LuLu Hypermarket and Carrefour |
|  | 1. In the past month, how often did you drink sugary drinks/soft drinks (e.g. Coke, Pepsi, 7up, Sports drink)? | - Never - 1 time per month - 2-3 times per month - 1 time per week - 2 times per week - 3-4 times per week - 5-6 times per week - 1 time per day - 2 times per day - 3 times or more per day |
|  | 1. In the past month, on average, how often did you order at-home delivery from fast food restaurants? | - Never - 1 time per month - 2-3 times per month - 1 time per week - 2 times per week - 3-4 times per week - 5-6 times per week - 1 time per day - 2 times per day - 3 times or more per day |
| 1. **Physical activity** | 1. During the last 7 days, on how many days did you do vigorous physical activities like heavy lifting, jogging, running, digging, aerobics, or fast bicycling? | - 1 day - 2 days - 3 days - 4 days - 5 days - 6 days - 7 days - No vigorous physical activities ***(Skip to question 34)*** |
|  | 1. How much time (in minutes) did you usually spend doing vigorous physical activities on one of those days? **(open-ended question)** |  |
|  | 1. During the last 7 days, on how many days did you do moderate physical activities like carrying light loads, bicycling at a regular pace, or doubles tennis? Do not include walking. | - 1 day - 2 days - 3 days - 4 days - 5 days - 6 days - 7 days - No moderate physical activities ***(Skip to submit)*** |
|  | 1. How much time (in minutes) did you usually spend doing moderate physical activities on one of those days? **(open-ended question)** |  |
