# Supplementary material for: Prevalence and factors associated with regular fast-food consumption among adults in the UAE: a cross-sectional study
Source: Public Health Nutr. 2025 Dec 16;29(1):e2. doi: 10.1017/S1368980025101560 (PMC12809606; doi:10.1017/S1368980025101560)
Supplement: Al Rajabi et al. supplementary material 2 — Al Rajabi et al. supplementary material [file S1368980025101560sup002.docx]

**Supplementary Table S2-** Unadjusted prevalence and adjusted odds ratios of regular fast-food consumption^a^ by sociodemographic and health-related characteristics among study participants who reported monthly household income (n=197).

|  | Unadjusted prevalence | | Regression model | |
| --- | --- | --- | --- | --- |
|  | **% [95% CI]** | ***P*-value^b^** | **AOR [95% CI]^c^** | ***P*-value^d^** |
| Overall | 48.2 [41.2-55.2] | NA | NA | NA |
| Sex |  |  |  |  |
| Women | 53.2 [45.4-61.0] | **0.008^*^** | 3.96 [1.15- 13.59] | **0.029^*^** |
| Men | 30.2 [18.1-44.9] |  | 1 [Reference] |  |
| Age (years) |  |  |  |  |
| 18 – 24 | 50.0 [42.0-58.0] | 0.386 | 1.11 [0.32-3.83] | 0.869 |
| 25 – 56 | 42.9 [29.7-56.8] |  | 1 [Reference] |  |
| Nationality |  |  |  |  |
| Emirati | 49.4 [42.1-56.8] | 0.353 | 1.47 [0.45-4.81] | 0.526 |
| Other nationalities | 39.1 [21.4-59.4] |  | 1 [Reference] |  |
| Emirate of residence |  |  |  |  |
| Abu Dhabi | 33.1 [25.9-41.0] | **<0.001^*^** | 1 [Reference] | **<0.001^*^** |
| Others^e^ | 93.9 [84.6-98.2] |  | 50.24 [13.04-193.56] |  |
| BMI (Kg/m^2^) |  |  |  |  |
| Underweight/Normal weight (<25.0) | 47.8 [39.5-56.2] | 0.857 | 1 [Reference] | 0.202 |
| Overweight/  Obese (≥25.0) | 49.2 [36.9-61.5] |  | 1.89 [0.71-5.01] |  |
|  |  |  |  |  |
| Marital status |  |  |  |  |
| Married/living with a partner | 45.5 [31.4-60.1] | 0.677 | 3.00 [0.90-10.01] | 0.073 |
| Single^f^ | 49.0 [41.2-56.9] |  | 1 [Reference] |  |
| Education |  |  |  |  |
| Less than Bachelor’s degree^g^ | 52.5 [42.8-62.0] | 0.221 | 1. 31 [0.50-2.55] | 0.767 |
| Bachelor’s degree or higher^h^ | 43.8 [34.1-53.7] |  | 1 [Reference] |  |
| Employment status |  |  |  |  |
| Employed | 44.4 [31.8-57.7] | 0.514 | 1.16 [0.36-3.80] | 0.802 |
| Unemployed^i^ | 49.7 [41.5-57.8] |  | 1 [Reference] |  |
| General Health |  |  |  | 0.250^k^ |
| Excellent | 32.4 [19.1-48.4] | 0.101 | 1 [Reference] |  |
| Very good/Good | 51.5 [43.0-60.0] |  | 2.33 [0.86-6.31] | 0.097 |
| Fair/Poor | 53.3 [35.9-70.2] |  | 1.89 [0.48-7.45] | 0.365 |
| Household income (AED/month) |  |  |  | 0.102^k^ |
| <10,000 | 54.8 [42.5-66.8] | 0.320 | 1.78 [0.56-5.60] | 0.325 |
| 10,000 – 80,000 | 46.8 [37.7-56.1] |  | 0.71 [0.23-2.13] | 0.535 |
| >80,000 | 37.5 [20.4-57.4] |  | 1 [Reference] |  |
| Physical activity^j^ |  |  |  |  |
| Low | 50.9 [41.8-59.9] | 0.382 | 1.31 [0.63-2.72] | 0.477 |
| Moderate-High | 44.6 [34.2-55.3] |  | 1 [Reference] |  |
| Smoking status |  |  |  |  |
| Current smoker | 56.3 [32.6-77.8] | 0.503 | 1.30 [0.29-5.87] | 0.736 |
| Former smoker/ Never smoker | 47.5 [40.3-54.8] |  | 1 [Reference] |  |
| Fruit and vegetable consumption |  |  |  |  |
| <2 servings/day | 47.4 [38.5-56.5] | 0.786 | 1 [Reference] | 0.771 |
| ≥2 servings/day | 49.4 [38.7-60.1] |  | 1.12 [0.53-2.33] |  |

^*^ Statistically significant

^a^ Consumed fast food ≥ 2 times/week based on the question: “*In the past month, on average, how many times did you go to a fast-food restaurant to eat?*”

^b^ Calculated by chi-square test comparing regular vs. non-regular fast-food consumers

^c^ Adjusted odds ratios from binary logistic regression model with regular fast-food consumption (≥2 times/week) as the dependent variable and all demographic, socioeconomic, and health-related characteristics as the covariates. Non-regular fast-food consumption was assigned as the reference level for the analysis.

^d^ Adjusted effect *p value*

^e^ Dubai, Sharjah, Ajman, Fujairah, Umm Al-Quwain, and Ras Al-Khaimah

^f^ Single includes: divorced, separated, widowed, and single/never married

^g^ Associate degree/college diploma, high school diploma, or less than a high school diploma

^h^ Bachelor’s degree or graduate degree (MSc, MBA, Ph.D., MD)

^i^ Unemployed includes: unemployed, retired, and student

^j^ Participants were categorized into low physical activity (<600 total MET- minutes/week) vs. moderate-to-high physical activity (≥600 total MET- minutes/week).

^K^ Overall *p-*value

Abbreviations: CI, confidence interval; AOR, adjusted odds ratios.
